# Physiological, Biochemical, and Yield Responses of Linseed (*Linum usitatissimum* L.) in α-Tocopherol-Mediated Alleviation of Salinity Stress

**DOI:** 10.3389/fpls.2022.867172

**Published:** 2022-06-03

**Authors:** Abdullah, Athar Mahmood, Safura Bibi, Maria Naqve, Muhammad Mansoor Javaid, Muhammad Anjum Zia, Abdul Jabbar, Wasi Ud-Din, Kotb A. Attia, Naeem Khan, Abdullah A. Al-Doss, Sajid Fiaz

**Affiliations:** ^1^Department of Botany, University of Agriculture, Faisalabad, Pakistan; ^2^Department of Agronomy, University of Agriculture, Faisalabad, Pakistan; ^3^Department of Agronomy, College of Agriculture, University of Sargodha, Sargodha, Pakistan; ^4^Department of Biochemistry, University of Agriculture, Faisalabad, Pakistan; ^5^Department of Biochemistry, College of Science, King Saud University, Riyadh, Saudi Arabia; ^6^Department of Agronomy, Institute of Food and Agricultural Sciences, University of Florida, Gainesville, FL, United States; ^7^Biotechnology Lab, Plant Production Department, College of Food and Agriculture Sciences, King Saud University, Riyadh, Saudi Arabia; ^8^Department of Plant Breeding and Genetics, The University of Haripur, Haripur, Pakistan

**Keywords:** enzymatic antioxidants, α-tocopherol, foliar application, saline, linseed

## Abstract

Exogenous application of antioxidants can be helpful for plants to resist salinity, which can be a potentially simple, economical, and culturally feasible approach, compared with introgression and genetic engineering. Foliar spraying of alpha-tocopherol (α-tocopherol) is an approach to improve plant growth under salinity stress. Alpha-tocopherol acts as an antioxidant preventing salinity-induced cellular oxidation. This study was designed to investigate the negative effects of salinity (0 and 120mM NaCl) on linseed (*Linum usitatissimum* L.) and their alleviation by foliar spraying of α-tocopherol (0, 100, and 200mg L^−1^). Seeds of varieties “Chandni and Roshni” were grown in sand-filled plastic pots, laid in a completely randomized design in a factorial arrangement, and each treatment was replicated three times. Salinity significantly affected linseed morphology and yield by reducing shoot and root dry weights, photosynthetic pigment (Chl. *a*, Chl. *b*, total Chl., and carotenoids) contents, mineral ion (Ca^2+^, K^+^) uptake, and 100-seed weight. Concomitantly, salinity increased Na^+^, proline, soluble protein, peroxidase, catalase, and superoxide dismutase activities in both varieties. Conversely, the growth and yield of linseed varieties were significantly restored by foliar spraying of α-tocopherol under saline conditions, improving shoot and root dry matter accumulation, photosynthetic pigment, mineral ion, proline, soluble protein contents, peroxidase, catalase, superoxide dismutase activities, and 100-seed weight. Moreover, foliar spray of α-tocopherol alleviated the effects of salinity stress by reducing the Na^+^ concentration and enhancing K^+^ and Ca^2+^ uptake. The Chandni variety performed better than the Roshni, for all growth and physiological parameters studied. Foliar spray of α-tocopherol (200mg L^−1^) alleviated salinity effects by improving the antioxidant potential of linseed varieties, which ultimately restored growth and yield. Therefore, the use of α-tocopherol may enhance the productivity of linseed and other crops under saline soils.

## Introduction

*Linseed (Linum usitatissimum)* is an annual herb of the Linaceae family, widely grown in the Mediterranean region due to its edible oil, durable fiber, food, and medicinal properties (Qayyum et al., 2019; Song et al., 2020). An increase in flex demand is due to its high protein, lignan, fiber, and linolenic acid-rich oil contents (Povkhova et al., 2021). Consumption of flax seeds or flaxseed oil reportedly improved cardiovascular health and proven beneficial for cancer, inflammatory diseases, and neurological and hormonal disorders (Ghobadi-Namin et al., 2020). Recent reports indicated that about 20% of total cultivated and 33% of irrigated lands are afflicted by saline conditions at present and more than 50% of the arable land would be affected by salts by the year 2050 (Dubey et al., 2020). Salinity stress negatively affects growth by the reduction in height, deterioration of the product quality and crop yields (Rahneshan et al., 2018), and physiological processes, such as a decrease in water uptake, chlorophyll content, and nutrient contents (Khataar et al., 2018; Hernandez, 2019). Currently, 1,125 million ha of lands are salt-affected, of which ~76 million ha are affected by salinization and sodification around the world. Due to salinization, soil degradation is a major limitation for agricultural productivity worldwide (Hossain, 2019). In Pakistan, the area affected by salinity is approximately 6.3 Mha of a total of 22 Mha of cropland (GOP, 2020); furthermore, ensuring global food security is rapidly becoming a major challenge because salinization already affects one-third of the total arable land worldwide (Tahir et al., 2018). Salinity stress has reduced the productivity of 6 Mha of agricultural land by 0.02 to 0.04 Mha per year (Lalarukh and Shahbaz, 2018).

Salinity reduces plant yield by interfering with morphological and biochemical functions (Arif et al., 2020) through the impairment of cellular metabolic processes, thereby reducing photosynthetic pigment contents, biomass accumulation, plant height, and ultimately, yield (Naqve et al., 2018; Riaz et al., 2019; Noreen et al., 2021). Significant structural and functional changes in the photosynthetic processes under salt stress are connected with changes in the structure of the thylakoid membrane, photosystem II (PSII) complex, photosynthetic electron transport chain, and a decrease in the photosynthetic activity. Moreover, the influence of salt stress has been shown on the interaction between QA and plastoquinone (PQ), as well as on PSI antenna size (Abdelgawad et al., 2018; Stefanov et al., 2021). Further, soil salinity disrupts plant-water relations by reducing soil and leaf water potential, thereby causing osmotic stress (Navada et al., 2020). In addition, ion toxicity is caused by the aggregation of Na^+^ions and the reduced uptake and accumulation of Ca^2+^ and K^+^ (Isayenkov and Maathuis, 2019), which in turn affects plant physiology and morphology (Lalarukh and Shahbaz, 2018). Increased cytosolic Ca^2+^ activated the SOS_2_-SOS_3_ protein kinase complex, which phosphorylates and stimulates the activity of SOS_1_ (PM Na^+^/H^+^ antiporter) and OsCPK12 and controls the detoxification of ROS by upregulating OsAPX2 and OsAPX8. Several calmodulins (CaM) and CaM-like (CML) proteins were found to be related to the osmotic and salt tolerance in plants (Chen et al., 2021).

Plants have evolved an antioxidant system that consists of enzymatic and non-enzymatic processes to counter the accumulation of salinity-induced reactive oxygen species (ROS) (Zainab et al., 2021). In addition, foliar spraying of antioxidant compounds can help to reduce the negative impact of high salt contents in plants (Naqve et al., 2021a). In addition to plant salinity tolerance and exclusion of toxic ions, plants can cope with salt-induced injury through the exogenous application of a variety of antioxidants, osmolytes, and plant hormones. Such treatments may be applied as foliar spraying or pre-sowing seed treatment; alternatively, they may be applied to the growth medium (Hasanuzzaman et al., 2020). Tocopherol (α, β, γ, or δ) is a natural, highly liposoluble antioxidant produced by green photosynthetic organisms (Chen et al., 2016; Naseer et al., 2020), which effectively protects biological membranes from salinity-induced oxidation (Naqve et al., 2021b). α-Tocopherol restores plant growth by detoxifying singlet oxygen species and peroxides, thereby shielding photosystems, and inhibiting lipid peroxidation under saline conditions (Zandi and Schnug, 2022). Maintaining membrane and organelle integrity is closely correlated with ROS scavenging capacity under salt stress (Hajihashemi et al., 2020). The resulting reduction of ROS levels protects chloroplasts from heat dissipation; specifically, α-tocopherol quinone inhibits photoexcitation of the photosynthetic machinery (Hu et al., 2020; Shanshan et al., 2020). In addition, α-tocopherol increases chlorophyll and carotenoid contents (Lalarukh et al., 2022) and regulates protein content, sugar level, and yield. It also improves antioxidant enzyme activities as well as the mineral ion (Ca^2+^ and K^+^) and proline contents (Sadiq et al., 2019). Overall, as an antioxidant, α-tocopherol prevents the formation of ROS and the proliferation of free radical reactions (Naqve et al., 2021c). Salinity causes severe economic losses to farmers in developing countries. Thus, there is an imperative need for agricultural experts to introduce and popularize cost-effective strategies for salinity damage remediation. The exogenous application of naturally synthesized α-tocopherol compound is one such approach. Therefore, this study aimed (i) to explore the impact of spraying two linseed varieties with α tocopherol to alleviate salinity's adverse effect on the growth and yield and (ii) to investigate the effect of the foliar spray of α-tocopherol on antioxidant-related biochemical traits of linseed plants grown under saline conditions.

## Materials and Methods

This research was performed at UAF Postgraduate Agricultural Research Station (Latitude: 31.383721 and Longitude: 72.989998) to study the influence of α-tocopherol (foliar spray) on growth and physio-biochemical traits of linseed plants under saline regimes. For this, plastic pots of diameter 24 cm and a depth of 30 cm having river sand (10 kg) were used. The varieties “Chandni and Roshni” seeds were taken from Ayub Agriculture Research Institute, Faisalabad, and 10 seeds were sown per pot, which afterward were thinned to six plants. Sand-filled plastic pots were laid in a completely randomized design (CRD) in a factorial arrangement with three replications. At the 3-week-old plants stage, Hoagland's nutrient solution (full strength) and two salt levels (0 and 120mM NaCl) were applied to each pot. The salt concentration of 120mM was maintained in aliquot parts of 60mM to prevent salt shock. Foliar application of α-tocopherol (0, 100, and 200mg L^−1^) concentration was sprayed on 36 days old plants. Tween-20 (0.1%) was applied as a surfactant to enhance the absorbance of the solution. The linseed plants in each pot provided 50ml of each level of α-tocopherol concentration (0, 100, and 200mg L^−1^). Plants were sprayed with α-tocopherol soon after sunset to prevent solution evaporation. After 21 days of foliar spray, three plants were carefully uprooted from each pot for data collection of shoots and roots. Plants were kept in an oven at 65°C and data for dry biomass was recorded. The physiological and mineral nutrients were recorded according to standard protocols. At maturity, plants were collected and sun-dried for measuring the weight of 100 seeds.

### Photosynthetic Pigments

The methods of Arnon (1949) and Davies (1976) were followed the determination of chlorophyll and carotenoid contents. A 0.1-g crushed fresh leaves has been placed in sterilized plastic bottles containing 10ml of 80% acetone solution. These bottles were kept in dark overnight at room temperature and read by using a spectrophotometer at 663, 645, and 480 nm.

### Nutrients Analysis

The acid digestion method proposed by Wolf (1982) was used to determine nutrient contents. In the digestion flask, 0.1 g of dried plant material was digested overnight at room temperature in 2mL H_2_SO_4_ and 1mL of H_2_O_2_ and was heated at 250°C until fumes appeared and the material converted colorless. Then, it was filtered and made its volume up to 50mL by distilled water. The mineral ions were measured using a flame photometer.

### Biochemical Traits

#### Superoxide Dismutase

For the measurement of SOD activities, Giannopolitis and Ries's (1977) procedure was adopted. The mixture was set in cuvettes by adding the specified amounts of phosphate buffer, H_2_O, Triton-X, L-methionine, NBT, enzyme extract, and riboflavin, and then the cuvettes were placed below a fluorescent lamp for 15 min and measured at 560 nm.

#### Catalase and Peroxidase

The leaf sample (0.5 g) was standardized in K_2_HPO_4_ buffer (50mM). The mixture was prepared by mixing potassium phosphate buffer (pH 7.0), guaiacol (20mM), H_2_O_2_ (40mM), and 0.1ml enzyme extract to approximate POD action. Absorbance at 470 nm was read for 20 s. The amount of CAT action examined was prepared by combining (50mM) phosphate buffer (pH 7.0), (5.9mM) H_2_O_2_, and (0.1ml) enzyme extract. After every 20 s, the absorbance at 240 nm was read by using the Chance and Maehly (1955) technique.

#### Proline

Bates et al. (1973) technique was used for estimating the proline content. A 0.5-g shoot material was taken and standardized with 10ml sulfosalicylic acid; 2.0ml of extract, glacial acetic acid, and ninhydrin acid were mixed with 4.0ml toluene and read at 520 nm by spectrophotometer.

#### Total Soluble Proteins

Fresh leaves material was ground in a chilled environment with KH_2_PO_4_ buffer, and the extract (5mL) was standardized with (1ml) Bradford Dye and (0.1 N) HCl and read at 595 nm using a spectrophotometer (Bradford, 1976).

#### Statistical Analysis

Analysis of variance (ANOVA) of data was calculated for all studied parameters using Statistix 8.1 software. Treatment comparison was made using Least Significance Difference (LSD) at a probability level of 5% (Steel et al., 1997).

## Results

### Growth Traits

**Figure 1:**
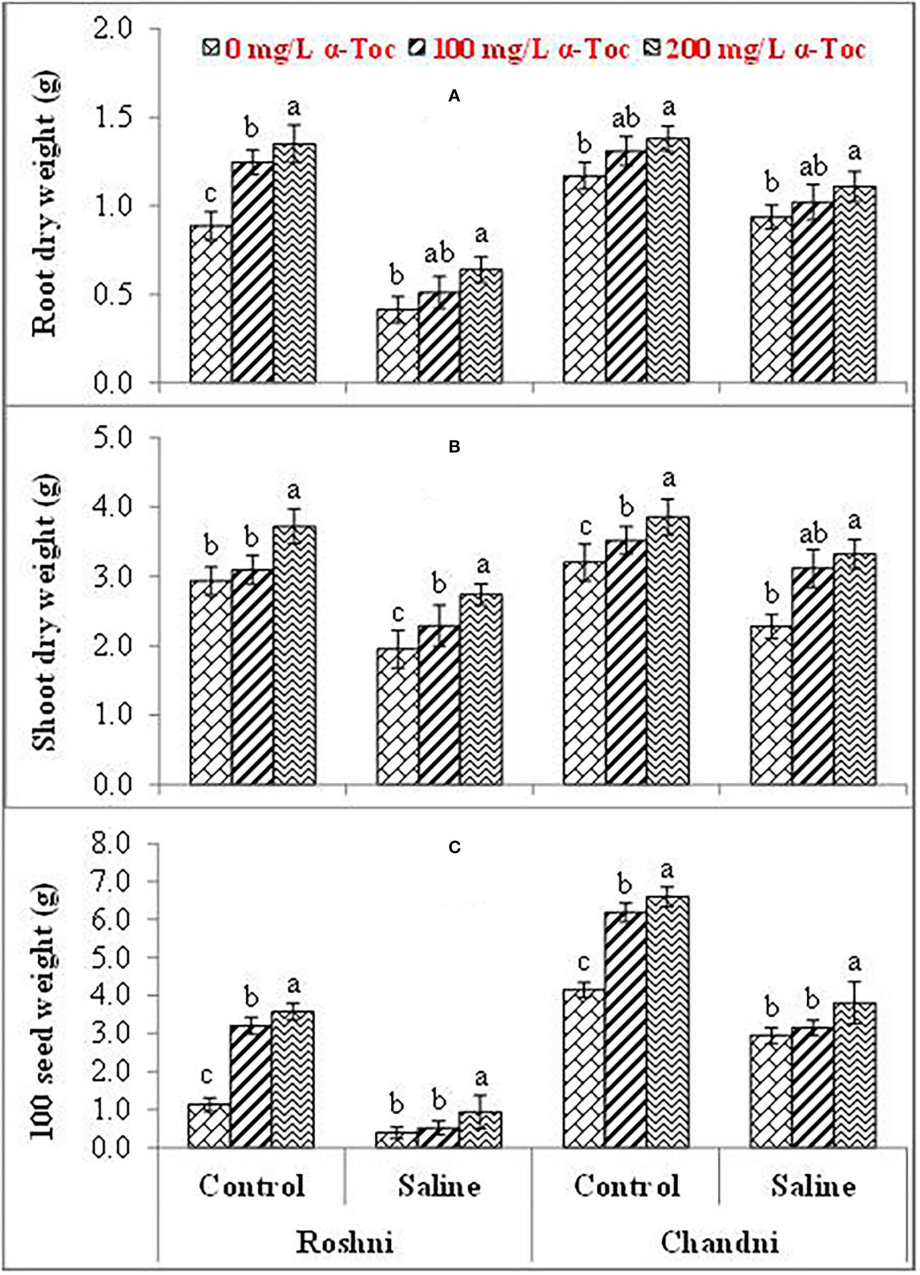
Root dry weight **(A)**, Shoot dry weight **(B)**, and 100-seed weight **(C)** of linseed varieties (Roshni and Chandni) sprayed with different levels of α-tocopherol under saline conditions. Values represent means ± S.D. Significant differences in row spacing were measured by the least significant difference (LSD) at *p* > 0.05 and indicated by different letters.

Salinity had a significant (*P* ≤ 0.05) impact on the shoot and root dry weight and 100-seed weight in both linseed tested varieties. Mean square values revealed that at 120mM NaCl concentration, variety Chandni performed better than that of variety Roshni in terms of the percent reduction in root and shoot dry weight and 100-seed weight. Furthermore, the interaction between variety and salinity was significant (*P* ≤ 0.05), while the interactions of α-tocopherol and salinity and α-tocopherol and variety were non-significant. Salt stress significantly reduced the growth attributes in both linseed varieties. Under salt stress, foliar spraying of α-tocopherol (100 and 200mg L^−1^) remarkably enhanced shoot and root dry weights and 100-seed weight in both linseed varieties. Variety Chandni showed more growth in terms of dry weights of the shoot, root, and seed as compared to that in variety Roshni. Salinity reduced the growth significantly while a foliar spray of α-tocopherol (200mg L^−1^) enhanced the seed, shoot, and root dry weight under saline conditions (Table 1 and Figures 1A–C).

**Table 1:** Analysis of variance (mean squares) for growth and physiological traits of two different linseed (*Linum usitassimum* L.) varieties (Roshni and Chandni) treated with α-tocopherol as foliar spray under saline and non-saline conditions.

**Source**	**df**	**RDW**	**SDW**	**100 Seed Wt**.	**Chl. *a***	**Chl. *b***	**Total Chl**.	**Car**.
Var	1	0.8836^***^	1.63840^***^	72.9601^***^	8.457^***^	1.335 ^***^	4.303^***^	0.04804^**^
Toc	2	0.22114^***^	2.00088^***^	7.8511^***^	4.558^***^	1.952^***^	1.246^***^	0.22325^***^
Sal	1	1.84054^***^	5.35151^***^	42.8807^***^	1.062^***^	6.898^***^	3.462^***^	0.84186^***^
Var × Toc	2	0.01923^ns^	0.09003^ns^	0.0235^ns^	1.873^ns^	4.270^**^	9.146^*^	0.00108^ns^
Var × Sal	1	0.31734^***^	0.20854^*^	0.2320^ns^	4.882^***^	2.667^***^	1.476^***^	0.00041^ns^
Toc × Sal	2	0.02241^ns^	0.09391^ns^	3.3070^***^	3.615^**^	3.349^*^	6.806^*^	0.06052^***^
Var × Toc × Sal	2	0.01034^ns^	0.03414^ns^	0.0186^ns^	3.615^**^	3.349^*^	6.806^*^	0.00529^ns^
Error	2	0.00677	0.04755	0.0813	1.209	1.546	1.955	0.00286
Var (LSD 0.05%)		0.0569	0.150	0.1971	0.59	0.11	0.14	0.037
Sal (LSD 0.05%)		0.0569	0.150	0.1971	0.59	0.11	0.14	0.037
α-Toc (LSD 0.05%)		0.0697	0.1846	0.2414	1.07	0.55	1.57	0.045

*, **, and *** *= significant at 0.05, 0.01, and 0.001 levels respectively, ns, non-significant; Var, Varieties; Sal, Salinity; α-Toc, Alpha tocopherol; RDW, Root dry weight; SDW, Shoot dry weight; 100-Seed wt., Seed weight; Chl. a, Chlorophyll a; Chl. b, Chlorophyll b; Total Chl., Total Chlorophyll; Car., Carotenoids*.

### Photosynthetic Pigments

**Figure 2:**
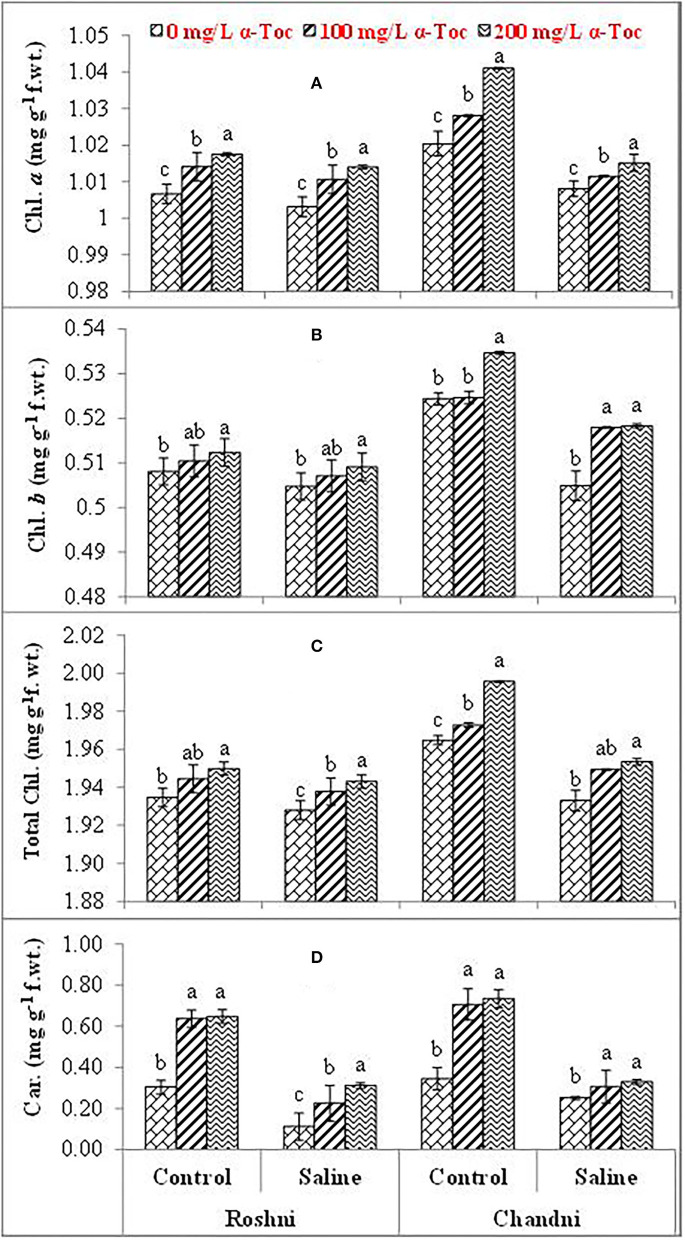
Chl. *a* **(A)**, Chl. *b* **(B)**, Total Chl. **(C)**, and Car. **(D)** of linseed varieties (Roshni and Chandni) sprayed with different levels of α-tocopherol under saline conditions. Values represent means ± S.D. Significant differences in row spacing were measured by the least significant difference (LSD) at *p* > 0.05 and indicated by different letters.

Salinity reduced the leaf chlorophyll *a, b*, and total chlorophyll and carotenoid concentrations in both linseed varieties. Mean square values revealed that interaction between salinity and variety was significant at *P* ≤ 0.001 for chlorophyll *a* and *b*, and total chlorophyll while salinity × tocopherol interaction was significant at *P* ≤ 0.01, *P* ≤ 0.05, *P* ≤ 0.001 for chlorophyll *a*, chlorophyll *b*, and total chlorophyll and carotenoids, respectively. Under saline conditions, variety Chandni had more pigment content than that of variety Roshni. The 200mg L^−1^ α-tocopherol treatment was more efficient in enhancing the attributes of both varieties under stress conditions. Foliar application of α-tocopherol significantly increased chlorophyll and carotenoid contents in salt-stressed plants. Of all α-tocopherol concentrations tested, 200mg L^−1^ was more effective for improving the attributes of salinity-treated plants of both varieties (Table 1 and Figures 2A–D).

### Ionic Content

**Figure 3:**
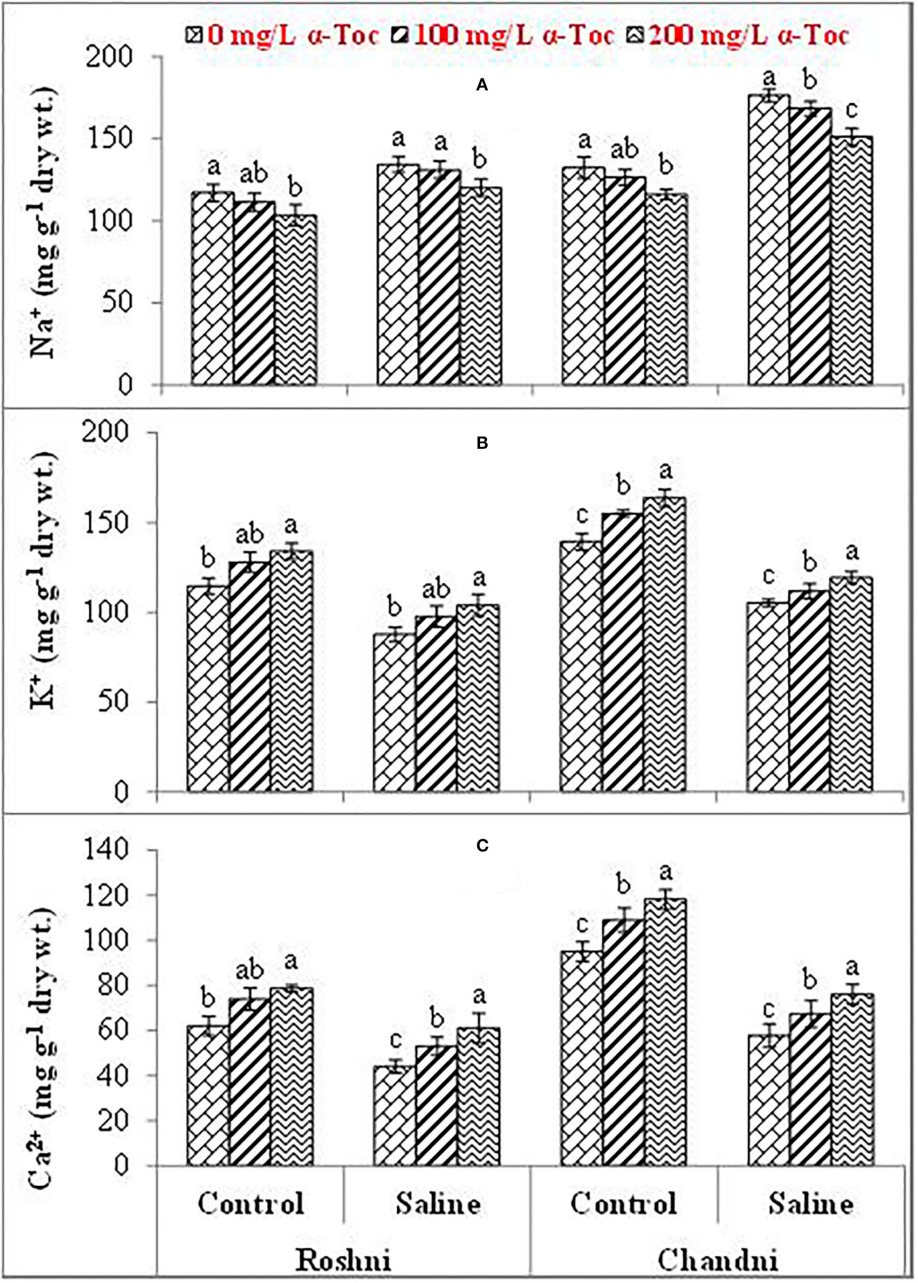
Na^+^ **(A)**, K^+^ **(B)**, and Ca^2+^ **(C)** of linseed varieties (Roshni and Chandni) sprayed with different levels of α-tocopherol under saline conditions. Values represent means ± S.D. Significant differences in row spacing were measured by the least significant difference (LSD) at *p* > 0.05 and indicated by different letters.

Mean square values for ionic content indicated that the interaction between variety and salinity was significant (P≤0.001), while variety×tocopherol interaction was non-significant. The Ca^2+^ and K^+^ contents decreased significantly in both linseed varieties, whereas the concentration of Na^+^ ions increased under the saline regime. In addition, Na^+^ accumulation varied remarkably in both linseed varieties. Foliar spraying with α-tocopherol altered the shoot Ca^2+^, K^+^, and Na^+^ ion contents in both linseed varieties. The Chandni variety showed higher contents of these ions than the Roshni variety. A spray of α-tocopherol remarkably altered the shoot Ca^2+^ and K^+^ contents in both linseed varieties under salt stress. In addition, foliar spraying with α-tocopherol significantly reduced Na^+^ contents in both linseed varieties (Table 2 and Figures 3A–C).

**Table 2:** Analysis of variance (mean squares) for ionic status, osmolytes, and antioxidant traits of two different linseed (*Linum usitassimum* L.) varieties (Roshni and Chandni) treated with α-tocopherol as foliar spray under saline and non-saline conditions.

**Source**	**df**	**Ca^**2+**^**	**K^**+**^**	**Na^**+**^**	**TSP**	**Proline**	**CAT**	**POD**	**SOD**
Var	1	5,650.03^***^	4,117.4^***^	5,877.78^***^	23.33^***^	46,735.23^***^	28.0723^***^	53.1198^***^	318.266^***^
Toc	2	1,067.53^***^	1,044.1^***^	945.03^***^	38.19^***^	14,336.62^***^	68.8764^***^	44.4943^***^	14.578^***^
Sal	1	7,891.36^***^	10,920.3^***^	7,627.11^***^	35.09^***^	11,653.20^***^	29.2501^***^	13.2375^***^	112.431^***^
Var × Toc	2	11.36^ns^	3.1^ns^	35.86^ns^	0.650^***^	316.55^***^	0.5658^ns^	0.2560^*^	0.916^ns^
Var × Sal	1	1,034.69^***^	294.7^***^	1,133.44^***^	0.045^***^	0.5136^**^	0.9312^*^	2.7060^***^	7.200^**^
Toc × Sal	2	10.19^ns^	42.3^***^	24.19^ns^	0.091^***^	534.76^***^	0.4155^ns^	12.1447^***^	0.345^ns^
Var × Toc × Sal	2	5.03^ns^	11.4^**^	13.03^ns^	0.0025^***^	0.625^***^	0.9361^*^	0.4341^**^	0.184^ns^
Error	2	19.98	3.1	24.66	0.007	118,652	0.1861	0.0638	0.605
Var (LSD 0.05%)		3.09	1.2178	3.43	3.05	23.70	0.2983	0.1746	0.5375
Sal (LSD 0.05%)		3.09	1.2178	3.43	3.05	23.70	0.2983	0.1746	0.5375
α-Toc (LSD 0.05%)		3.7847	1.4915	4.2041	3.744	29.02	0.3653	0.2138	0.6583

*, **, and *** * = significant at 0.05, 0.01, and 0.001 levels, respectively, ns, non-significant; Var, Varieties; Sal, Salinity; α-Toc, Alpha tocopherol; Ca^2+^, Calcium ion; K^+^, Potassium ion; Na^+^, Sodium ion; TSP, Total soluble proteins; CAT, Catalase; POD, Peroxidase; SOD, Superoxide dismutase*.

### Biochemical Traits

#### Enzymatic Antioxidant

**Figure 4:**
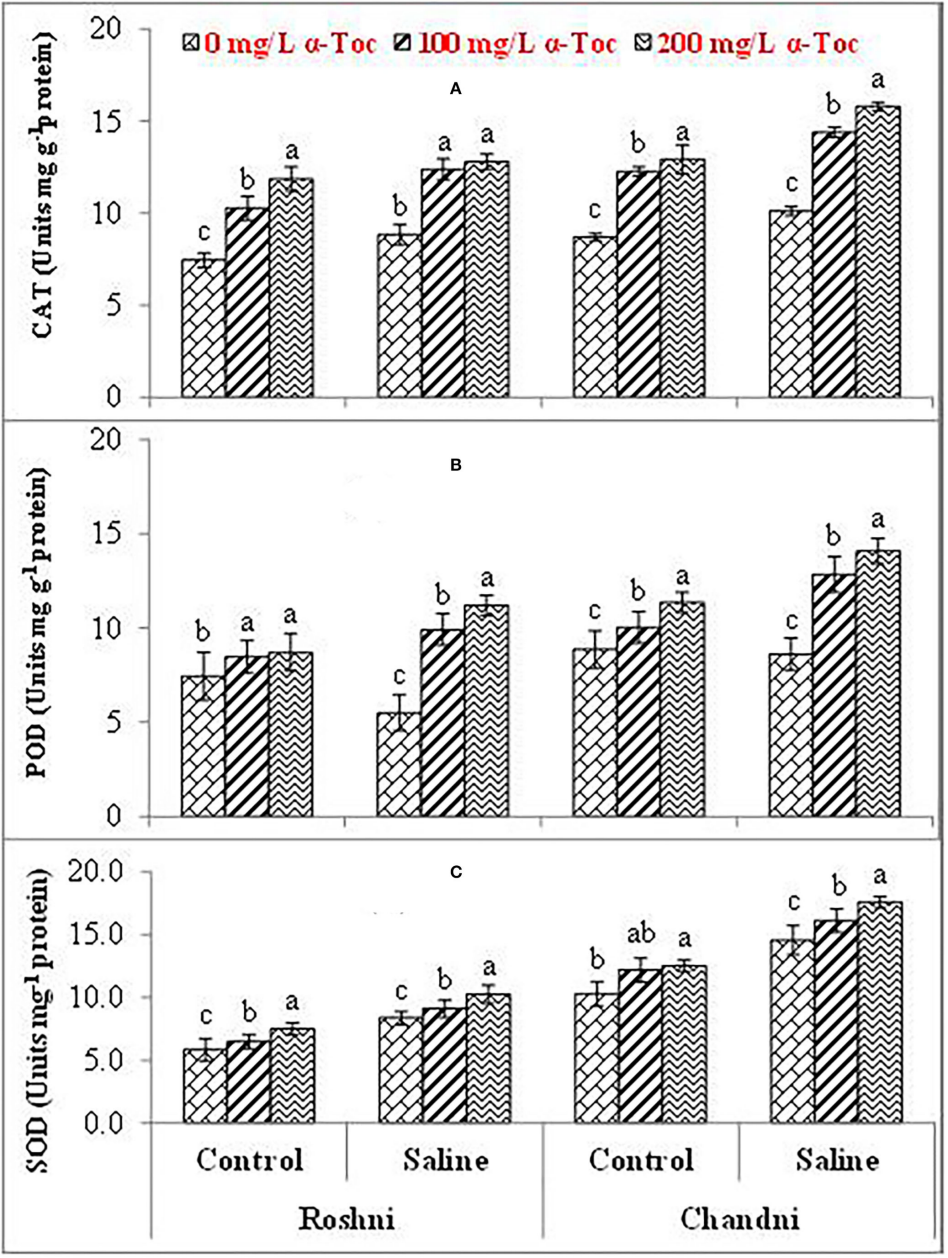
CAT **(A)**, POD **(B)**, and SOD **(C)** of linseed varieties (Roshni and Chandni) sprayed with different levels of α-tocopherol under saline conditions. Values represent means ± S.D. Significant differences in row spacing were measured by the least significant difference (LSD) at *p* > 0.05 and indicated by different letters.

The mean square values exhibited a significant difference in enzymatic antioxidants (*P* ≤ 0.001) with variety × salinity interaction; however, a non-significant difference was observed with variety × tocopherol and tocopherol × salinity interactions for CAT and SOD activities. The enzymatic antioxidant activity increased significantly in both linseed varieties under salinity stress. Variety Chandni showed higher antioxidant enzymatic activities (CAT, POD, and SOD) than the variety Roshni under saline conditions. Furthermore, variety Chandni showed higher SOD, POD, and CAT activities than variety Roshni when α-tocopherol was used as a foliar spray under salinity conditions. Foliar application of 200mg L^−1^ α-tocopherol remarkably enhanced enzymatic antioxidant activities compared to 100mg L^−1^ in high-salinity regimes (Table 2 and Figures 4A–C).

#### Organic Osmolytes

**Figure 5:**
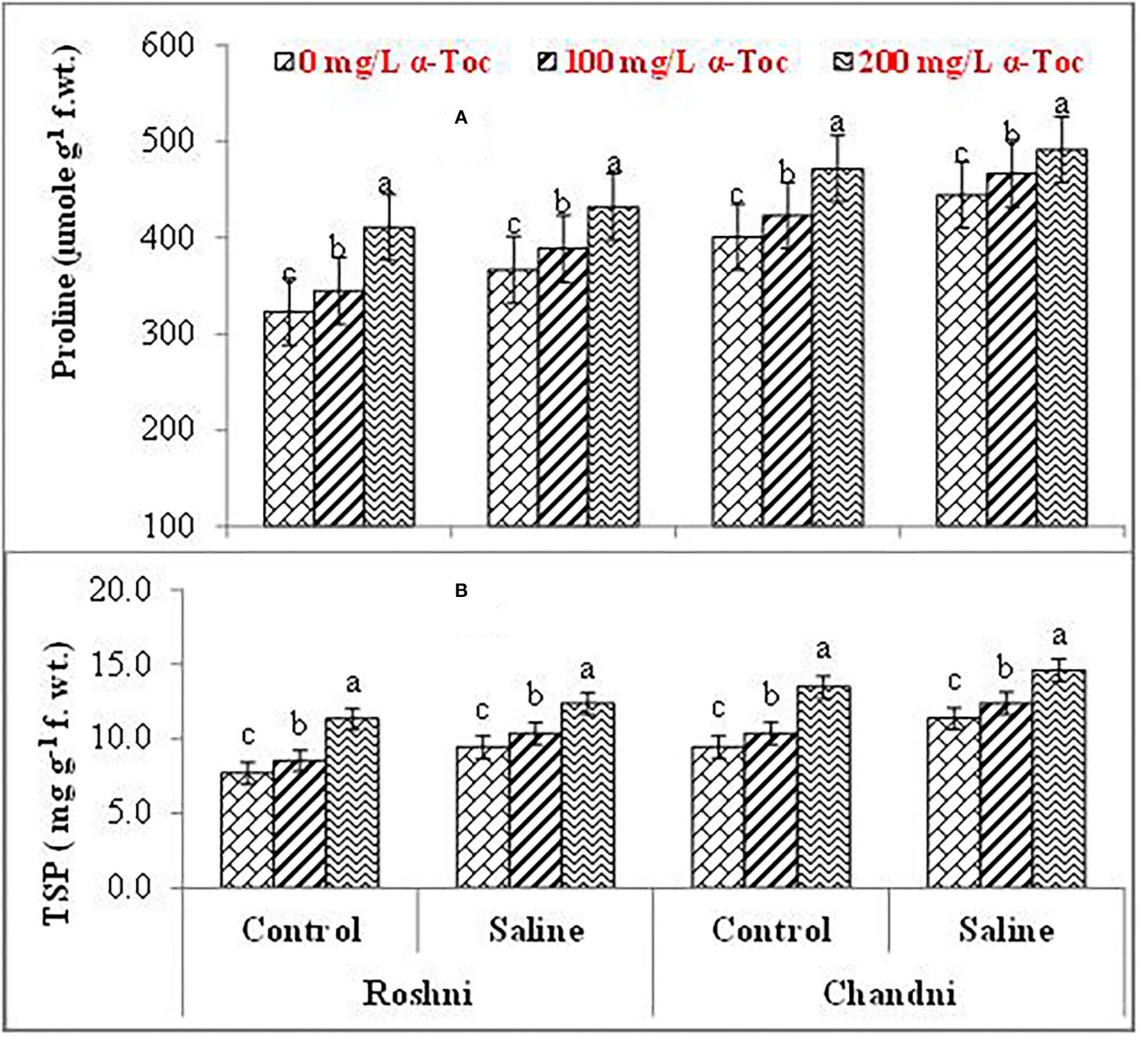
Proline **(A)** and TSP **(B)** of linseed varieties (Roshni and Chandni) sprayed with different levels of α-tocopherol under saline conditions. Values represent means ± S.D. Significant differences in row spacing were measured by the least significant difference (LSD) at *p* > 0.05 and indicated by different letters.

Mean square values exhibited that variety × salinity, varieties × tocopherol, and tocopherol × salinity interactions showed significant differences (*P* ≤ 0.001) for proline and soluble protein content. In terms of osmolyte contents, linseed varieties differed significantly from the variety Chandni and exhibited higher osmolyte content than the variety Roshni. Alpha-tocopherol spray at 200mg L^−1^ enhanced proline and soluble protein contents to a greater extent compared to that of 100mg L^−1^ in both linseed varieties. Similarly, under salinity conditions, foliar spraying of α-tocopherol (200mg L^−1^) considerably enhanced proline and soluble protein contents in comparison to 100mg L^−1^ of α-tocopherol in both varieties (Table 2 and Figures 5A,B).

## Discussion

Salinity stress can damage the physiological mechanisms of plants (Kirsch et al., 2019). Salinity stress has become a major threat to agriculture and it has been estimated that the yield of economically significant crops can reduce by 50% if the current rate of salinization remains to continue (Mbarki et al., 2020). The visual symptoms of salinity stress include reduced growth. In this study, the biomass (root and shoot dry weight) decreased significantly with an increase in salinity in tested varieties of linseed. Salinity effects could be alleviated by employing shot-gun approaches, that is, exogenous application of vitamins, nutrients, and antioxidants. One of these antioxidants is α-tocopherol, which can be exogenously applied as it is produced by plants endogenously (Naqve et al., 2021b). In this study, reduction in linseed has been alleviated by foliar spray of α-tocopherol. This showed that α-tocopherol supplementation increases plant growth by stimulating various signaling factors involved in plant growth regulation. In the current investigation, a reduction in 100-seed weight has been observed in tested linseed varieties under salinity stress. Reduction in yield might be attributed to stunted growth, reduced rate of photosynthesis, and ionic stress under saline conditions.

The quantity and quality of photosynthetic pigments play a critical role in plants by carbon assimilation, thus making them extremely important to all components of the photosynthetic system. However, salinity stress disorganizes the plastid structure and ultimately disturbs the biosynthesis of chlorophyll and other photosynthetic pigments, including leaf chlorophyll *a* and *b*, total chlorophyll, and carotenoid concentration. In this study, salt-induced stress significantly reduced all these pigments in both the tested varieties of linseed. Foliar spray of α-tocopherol significantly enhanced chlorophyll *a* and *b*, total chlorophyll, and carotenoid contents, and 200mg L^−1^ spray was more effective in enhancing the concentration of the pigment. Altered pigment concentrations cause a reduction in photosynthesis rate, as the impact of salinity stress on photosynthesis may include the disruption of the reaction center of photosystem II (PSII) and the electron transport chain. α-tocopherol is synthesized in plastids and acts as an antioxidant to protect photosynthetic pigments and alleviate salinity-induced oxidative bursts (Naqve et al., 2021c). Thus, while protecting chloroplast structure and thylakoid membranes from photo-inhibition, α-tocopherol plays a crucial role in plant tolerance to high salinity conditions. Indeed, exogenously applied α-tocopherol treatment optimized carotenoid and chlorophyll content under salinity stress.

Salt stress imbalances the uptake of nutrients which ultimately disintegrate the membrane and ultrastructure of the cell. Consequently, salinity leads to osmotic and ionic stress (Singh et al., 2021). Further, a nutritional imbalance is a major problem induced by salinity stress. In this study, Na^+^ ion concentration was significantly increased in the tissues of both linseed varieties under salinity stress. Such elevated Na^+^ levels reportedly interrupt photosynthesis, metabolism, and antioxidant activities, resulting in low plant productivity. The amount of K^+^ and Ca^2+^ ions was significantly reduced in tested varieties of linseed under salinity stress. This nutritional imbalance under salinity conditions results in osmotic stress and ROS accumulation (Cambridge et al., 2017). The accumulation of Na^+^ caused by high salinity is preceded by a decrease in K^+^ concentration, indicating that Na^+^ and K^+^ ions are antagonistic. Salinity affects plant processes by imposing osmotic stress and destroying ionic and redox signaling (Singh et al., 2021).

K^+^ absorption is impaired by high salt (NaCl) levels in the soil and groundwater. In turn, a reduction in K^+^ uptake adversely affects water relations, enzymatic activities, and protein biosynthesis (Chokshi et al., 2017). In turn, calcium acts in signal transduction processes and provides membrane structural stability; however, toxic salt concentrations reduce Ca^2+^ uptake. Our data showed that salinity significantly reduced the content of Ca^2+^ and K^+^ ions and enhanced Na^+^ in both root and shoot tissues of both varieties in this study. However, a significant increase in Ca^2+^ and K^+^ ion content in shoots and roots of linseed plants upon foliar spraying with α-tocopherol enhanced K^+^ and Ca^2+^ uptake to maintain ionic balance and ionic homeostasis, thus adjusting physiological performance to the saline environment. Calcium plays a vital role in plants by providing structural stability and as a signaling molecule, but high concentrations of toxic salts reduced the uptake of Ca^2+^. Alpha-tocopherol supplementation helps lower NaCl ion content and enhances the uptake of K^+^ under abiotic stress and non-stressed conditions (Ahmed et al., 2021).

Higher concentrations of reactive oxygen species (ROS) are produced under salinity stress, causing tissue damage by oxidization of macromolecules and, consequently, reducing crop productivity. The production of antioxidant agents for ROS quenching is a common plant strategy to prevent or minimize salinity stress-induced injury (Gulcin, 2020). Such reduction is reportedly associated with the closing of stomata and results in severely reduced diffusion of CO_2_ into the leaf mesophyll in plants under saline conditions.

In addition, enzymatic antioxidants play a vital role in preventing salinity-induced damage due to ROS accumulation. Particularly, in this study, foliar spraying of α-tocopherol enhanced SOD, CAT, and POD enzyme activities considerably under salinity stress. These enzymes are well-known to be upregulated in coordination with α-tocopherol and help plants resist oxidative bursts induced by salinity (Hasanuzzaman et al., 2020). Thus, SOD activity quenches singlet oxygen species, while CAT and POD activities contribute to the efficient quenching of H_2_O_2_ (Ali et al., 2018). Thus, in addition to its ROS-quenching antioxidant effect, α-tocopherol supplementation helps to counter salinity-induced oxidative bursts by upregulating enzymatic antioxidant activities, such as CAT, POD, and SOD.

Soluble proteins are compatible solutes that increase under salinity stress. In this study, salinity stress significantly increased the total soluble protein content of linseed, and foliar spraying of α-tocopherol resulted in a significant increase in the total soluble protein content. Protein biosynthesis is aided by α-tocopherol. Similarly, proline is well known to accumulate in salinity-stressed plants to protect plant tissues against osmotic damage (Qayyum et al., 2020). To cope with osmotic stress, salt-stressed plants tend to accumulate compatible solutes, such as proline, that decrease the osmotic potential enhancing water absorption (Abdelgawad et al., 2018). Consistently, we observed an enhanced leaf proline content under salinity stress in tested varieties of linseed. As an antioxidant, proline protects plants from the harmful effects of salts by shielding the photosynthetic machinery and acting as an osmolyte. Consistently, the use of α-tocopherol as a foliar spray increased proline content, suggesting that α-tocopherol plays a defensive role against salinity-induced injury by promoting osmotic adjustment.

Increased levels of total soluble proteins are thought to boost SOD activity in plants and decrease the negative effects of ROS (Naz et al., 2019). Here, salinity significantly enhanced total soluble protein content in linseed plants, and a highly significant increase of total soluble protein was observed upon foliar spraying of α-tocopherol under salinity stress. Indeed, exogenous spraying of α-tocopherol and protein synthesis were positively correlated. Results of this study indicated that salinity stress increased leaf proline in the tested linseed varieties because proline accumulation and salinity stress-responsive proteins were positively correlated.

Plants are highly susceptible to abiotic and biotic stress conditions, which cause significant yield losses (Naz et al., 2018). In the experiments reported herein, a reduced number of seeds were produced under high salinity conditions. However, both in the control and salinity treatments, foliar application of α-tocopherol increased plant yield. A reduction in the uptake of essential nutrients was directly associated with low yield under salinity stress. Moreover, α-tocopherol spraying significantly enhanced plant yield under control and salinity conditions. Altogether, our data strongly indicated that the application of α-tocopherol enhanced the uptake of essential nutrients, greater chloroplast stability, and a reduction of oxidative stress, all of which were closely related to the observed increase in yield under salinity stress conditions.

## Conclusion

Foliar spray of α-tocopherol proved effective in the alleviation of salinity-induced damages in linseed by increasing growth, photosynthetic pigments, ionic contents, and biochemical traits possibly by protecting chloroplast due to its antioxidant potential. Among the tested linseed varieties, Chandni showed enhanced tolerance against salinity and 200mg L^−1^ α-tocopherol was more effective. Thus, this study points to the use of linseed variety Chandni to be grown in saline soils with foliar spray of α-tocopherol (200mg L^−1^) to enhance linseed production under field conditions. Foliar spray of α-tocopherol is also recommended to apply on other crops to alleviate salinity-induced damages.

## Data Availability Statement

The original contributions presented in the study are included in the article/supplementary materials, further inquiries can be directed to the corresponding authors.

## Author Contributions

A conceived the idea and conducted research. SB, MN, and AM carried out the investigation and helped in the writing of the original draft. MJ, MZ, AJ, and WU-D designed the data analysis and layout. KA, NK, and AA-D provided technical expertise. SF, AM, and KA helped in the revision and re-analysis of the data. All authors carefully read, revised, and approved the article for submission.

## Conflict of Interest

The authors declare that the research was conducted in the absence of any commercial or financial relationships that could be construed as a potential conflict of interest. The reviewer SF declared a shared affiliation with the author SF to the handling editor at the time of review.

## Publisher's Note

All claims expressed in this article are solely those of the authors and do not necessarily represent those of their affiliated organizations, or those of the publisher, the editors and the reviewers. Any product that may be evaluated in this article, or claim that may be made by its manufacturer, is not guaranteed or endorsed by the publisher.
